# *Poa annua* becomes the first weed to evolve resistance to indaziflam applied preemergence and early‐postemergence

**DOI:** 10.1002/ps.70214

**Published:** 2025-09-08

**Authors:** Joshua WA Miranda, Todd A Gaines, Marcelo L Moretti

**Affiliations:** ^1^ Department of Horticulture Michigan State University East Lansing MI USA; ^2^ Department of Horticulture Oregon State University Corvallis OR USA; ^3^ Department of Agricultural Biology Colorado State University Fort Collins CO USA

**Keywords:** annual bluegrass, cellulose biosynthesis inhibitor, herbicide resistance, temperature‐dependent herbicide response

## Abstract

**BACKGROUND:**

Herbicide resistance evolution is a major challenge in agriculture. *Poa annua* L., a globally distributed and genetically diverse weed, has repeatedly evolved resistance to multiple herbicide sites of action due to its genetic plasticity and rapid life cycle. Indaziflam is widely used for *P. annua* control in several agroecosystems, with reports of postemergence resistance and a few confirmed cases of resistance to preemergence application. This study investigated suspected resistance to indaziflam in *P. annua* accessions from Oregon hazelnut orchards.

**RESULTS:**

Whole‐plant dose–response assays confirmed indaziflam resistance in eight accessions ranging from 2.5‐ to 51‐fold relative to susceptible accessions, which had a lethal dose to 50% of the accession (LD_50_ ) of 1.0–3.4 g ha^−1^. Resistance increased with plant developmental stage and was most pronounced when indaziflam was applied early‐postemergence, with some accessions surviving two‐ to four‐fold the labeled rate in tree nut orchards (50–95 g ha^−1^). Field experiments confirmed reduced efficacy of preemergence and early‐postemergence indaziflam treatments up to 195 g ha^−1^. Indaziflam efficacy declined significantly across all accessions at air temperatures 9 °C:1 °C (day/night), with LD_50_ values increasing up to eight‐fold across accessions compared to 25 °C:12 °C (day/night). Enzyme inhibitor seed‐based assays with cytochrome P450 and glutatione transferase inhibitors did not reverse resistance, suggesting resistance pathways other than enhanced metabolism may be involved.

**CONCLUSION:**

This study provides the first confirmed cases of field‐evolved resistance to indaziflam applied both preemergence and early‐postemergence in *P. annua*. Resistance was most severe under cooler temperatures and postemergence use, highlighting environmental and developmental effects on indaziflam activity. © 2025 The Author(s). *Pest Management Science* published by John Wiley & Sons Ltd on behalf of Society of Chemical Industry.

## Graphical abstract

We report the first confirmed global case of *Poa annua* evolving resistance to indaziflam applied both preemergence and early‐postemergence. Resistance intensified under cool temperatures, was not reversed by metabolic inhibitors, and was often linked to multiple resistance, underscoring urgent needs for integrated weed management.

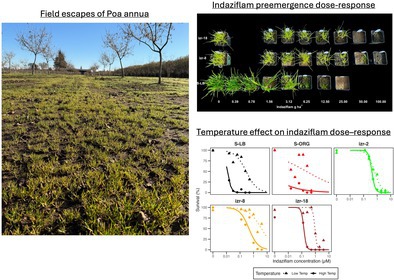

## 1 INTRODUCTION

Herbicide resistance is a major challenge in modern agriculture.^1^, ^2^ Since the widespread adoption of herbicides in the mid‐20th century, global dependence on a limited number of herbicide sites of action (SOA) has led to the repeated evolution of resistant weed biotypes across diverse agroecosystems.^2^, ^3^, ^4^ As of 2025, more than 534 unique cases of herbicide resistance have been confirmed globally, involving over 270 weed species, with resistance documented to 21 of the 31 currently recognized herbicide SOAs.^5^, ^6^

Among these species, *Poa annua* L. (annual bluegrass) is particularly problematic.^7^ It is a cosmopolitan winter weed known for its ecological plasticity and genetic adaptability. *Poa annua* thrives in managed turfgrass systems such as golf courses and athletic fields, as well as in orchards, vineyards, roadside margins, and other diverse agroecosystems.^7^, ^8^ Although primarily a winter annual, it can also persist as a short‐lived perennial in temperate climates.^9^, ^10^ This flexibility in life history allows *P. annua* to establish itself across a range of management regimes and environmental conditions. This species has rapid growth, prolific seed production, minimal seed dormancy, and continuous germination year‐round.^8^, ^9^, ^10^ From a genetic standpoint, *P. annua* is an allotetraploid (2*n* = 4*x* = 28),^11^ originating from interspecific hybridization between *Poa infirma* Kunth and *Poa supina* Schrader.^12^ *Poa annua* has evolved resistance to at least 12 distinct herbicide SOAs.^5^ Cases of multiple resistance, in which a single accession evolves resistance to two or more SOAs, are increasingly prevalent, with some accessions evolving resistance to five or even seven SOAs.^13^, ^14^

Indaziflam is an herbicide belonging to the Herbicide Resistance Action Committee (HRAC) Group 29 and is classified as a cellulose biosynthesis inhibitor (CBI).^15^ It is proposed to impair the trafficking and spatial organization of cellulose synthase complexes (CSCs) at the plasma membrane, disrupting cellulose microfibril deposition in the primary cell wall.^15^ This disruption impedes cell division and expansion during early seedling development, ultimately preventing emergence.^16^, ^17^, ^18^ Applied preemergence or early postemergence, indaziflam provides extended soil residual activity due to its lipophilic nature and strong binding to soil colloids, forming a persistent herbicide layer that suppresses seedling emergence for several months.^17^, ^18^, ^19^, ^20^ These characteristics make indaziflam particularly effective in perennial systems such as hazelnut orchards, where maintaining clean orchard floors is essential for harvest. However, prolonged soil residual activity creates recurrent selection pressure on successive generations of weed populations, especially in systems with limited herbicide diversity and continuous annual use.^21^ Moreover, the absence of a clearly defined indaziflam molecular target complicates proactive resistance monitoring and delays the development of diagnostic tools.

Indaziflam resistance has been documented, most commonly after postemergence applications,^14^ with only one confirmed case under preemergence conditions.^20^ In 2022, hazelnut growers in Oregon's Willamette Valley, USA, reported failures in controlling *P. annua* following preemergence indaziflam applications at 95 g active ingredient (a.i.) ha^−1^, labeled rate. These reports originated from orchards with a history of repeated indaziflam use over consecutive seasons, suggesting the emergence of resistance. Field visits confirmed the presence of mature, reproductive *P. annua* plants, with no other weed species present, a further indicative of herbicide resistance rather than misapplication.

This study aimed to (1) confirm and quantify resistance to indaziflam in *P. annua* accessions collected from hazelnut orchards, (2) assess the effect of temperature on resistance levels, (3) investigate metabolic‐based resistance using chemical inhibitors, (4) determine resistance to other herbicide SOAs, and (5) validate resistance under field conditions. This study presents the first confirmed case of preemergence and early‐postemergence indaziflam resistance in any weed species worldwide. These findings advance the understanding resistance risk associated with CBIs and offer valuable guidance for the management of *P. annua* in perennial production systems.

## 2 MATERIALS AND METHODS

### 2.1 Plant material and accession development

**Figure 1:**
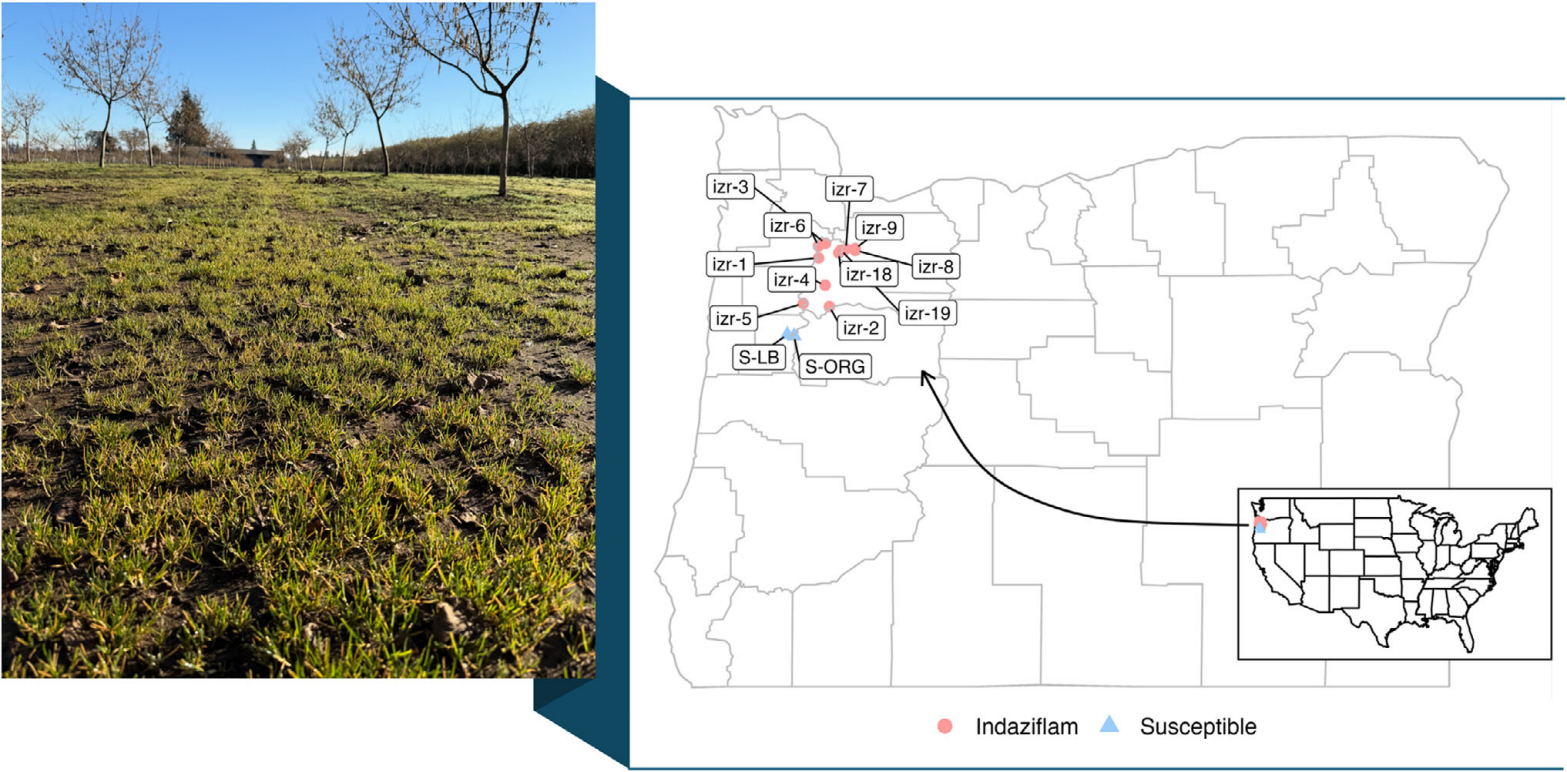
Field survey sites and geographic distribution of *Poa annua* accessions collected from Oregon's Willamette Valley. Red circles represent resistant accessions with survival to indaziflam (izr), and blue triangles represent susceptible reference accessions (S‐LB and S‐ORG). The photograph on the left shows a hazelnut orchard exhibiting poor *P. annua* control following indaziflam treatment.

Between spring and early summer of 2022, *P. annua* plants were collected from 11 hazelnut orchards across Oregon's Willamette Valley (Fig. 1) where industry members had reported control failures following indaziflam use. Plants were transplanted into a glasshouse at Oregon State University and maintained under controlled conditions (22 °C:18 °C day/night, 12‐h photoperiod). As plants reached the boot stage, each was isolated by pollen‐proof pollination bags (Mini Bag 3D M15.30.15; PBS International, Eastfield, UK) to prevent cross‐pollination and maintain genetic isolation. Seeds were harvested, cleaned, and stored under ambient conditions for 4 weeks.

Initial glasshouse screening was conducted to identify resistant accessions by applying 25 or 50 g ha^−1^ indaziflam (Alion®; Bayer CropScience, Research Triangle Park, NC, USA) to progeny from all 11 field‐collected accessions using a research sprayer (DeVries Manufacturing, Generation III, Hollandale, MN, USA) equipped with a single TP8003E nozzle (TeeJet Technologies, Glendale Heights, IL, USA), placed 45 cm above the canopy, and calibrated to deliver 187 L ha^−1^ of spray solution at 207 kPa. Eight of the 11 accessions survived and were selected for further studies. These accessions were advanced through two to three generations of single‐seed descent, to reduce genetic variability and establish stable accessions for phenotypic evaluation. For each generation, a single plant surviving after 25 or 50 g ha^−1^ indaziflam treatment was selected for self‐pollination and isolated using the same pollen‐proof bags; resultant seeds were harvested, and resultant progenies were propagated under identical conditions.

In addition, two susceptible accessions were included for comparative purposes: one collected from an organically managed research field with no history of herbicide use (S‐ORG), and another from a campus turfgrass area where indaziflam had not been applied (S‐LB). All progenies and accessions were maintained under similar glasshouse conditions and used in subsequent experiments.

### 2.2 General assay protocol

Two core assay protocols were used throughout this study: seed‐based and whole‐plant assays. Seed‐based assays provided a rapid, early‐detection method for resistance phenotyping. As demonstrated in previous studies, this method effectively identified resistant plants regardless of underlying resistance mechanisms.^22^ In contrast, whole‐plant assays assessed plant survival and growth after herbicide application under controlled glasshouse conditions, simulating field conditions for a more realistic evaluation of resistance. Standard protocols were followed unless otherwise noted in each experiment.

Seed‐based assays followed protocols adapted from Perez *et al*.^23^ Seeds were soaked for 6 h in distilled water or herbicide solution depending on the experiment. This pretreatment aimed to simulate early imbibition and initiate germination‐linked metabolic activity.^24^ Following soaking, seeds were rinsed with distilled water, gently dried on paper towels, and placed in 90‐mm Petri dishes lined with two layers of Whatman No. 1 filter paper, moistened with 4 mL of the test solution (distilled water or inhibitor). Each Petri dish, containing ten seeds, was considered an experimental unit. Plates were sealed with Parafilm and incubated at 22 °C with a 12 h:12 h light/dark cycle at a photon flux density of 300 μmol m^−2^ s^−1^, unless otherwise stated. Seedlings were visually evaluated 30 days after establishment of each experiment: those with radicle and/or coleoptile growth ≥ 1 cm were counted as ‘alive,’ while those without visible growth or with necrotic tissue were considered dead. Each experiment followed a completely randomized design with three biological replicates per treatment and was repeated twice.

Whole‐plant dose–response experiments were conducted using preemergence or postemergence applications to validate seed assay results. For both application timings, pots were filled with sterilized Willamette‐series silty clay loam soil. Indaziflam and other herbicides (Section 2.2.4 – multiple resistance screening) were applied with a research sprayer as described previously. Pots were maintained under glasshouse conditions at 22 °C:18 °C and 12‐h photoperiod, unless otherwise stated. Each experiment followed a completely randomized design with four biological replicates per treatment and was repeated twice.

#### 2.2.1 Dose–response bioassay

Eight resistant and two susceptible accessions were evaluated in seed assays by dose–response experiments. Seeds from each accession were soaked for 6 h in indaziflam solutions dissolved in distilled water at concentrations ranging from 0.01 to 4.0 μm. The concentration range was based on preliminary germination assays to span the full response curve, from minimal inhibition at low doses to complete inhibition at high doses. Control treatments used distilled water only.

For preemergence whole‐plant assays, dose–response experiments were conducted using the two susceptible accessions (S‐ORG and S‐LB) and the three most resistant accessions (izr‐2, izr‐8, izr‐18), as identified through initial seed‐based screening. Ten seeds per pot were sown into 0.5‐L plastic pots filled with sterilized Willamette‐series loam soil. Indaziflam was applied at 0, 6.25, 12.5, 25, 50, and 100 g ha^−1^. Additionally, the other five resistant accessions (izr‐1, izr‐4, izr‐5, izr‐9, izr‐19) were evaluated only at 25 and 50 g indaziflam ha^−1^ to estimate survival percentages (Table 1). Seeds were covered with 0.5 cm of soil and treatments applied immediately after sowing. Irrigation was withheld for 24 h following application as recommended by the manufacturer label to allow herbicide binding. Plant emergence was assessed 8 weeks after treatment; individuals were scored as ‘alive’ if cotyledons had emerged above the soil surface (i.e., germinated).

**Table 1:** *Poa annua* survival 8 weeks after preemergence application of indaziflam at 25 and 50 g ha^−1^ under glasshouse conditions

Accession	Plant survival (%)^†^	
	25 g indaziflam ha^−1^	50 g indaziflam ha^−1^
izr‐1	25 ± 15b	25 ± 15a
izr‐2	25 ± 15b	25 ± 15a
izr‐4	25 ± 15b	13 ± 11a
izr‐5	25 ± 15b	25 ± 15a
izr‐8	25 ± 15b	13 ± 11a
izr‐9	50 ± 17ab	13 ± 11a
izr‐18	25 ± 15b	13 ± 11a
izr‐19	63 ± 17a	13 ± 11a
S‐LB	0 ± 0c	0 ± 0b
S‐ORG	0 ± 0c	0 ± 0b

^†^ Values are shown as mean ± the standard error (*n* = 8). Different letters within the same column indicate significant differences at *P* < 0.05 (Tukey's HSD).

For the early‐postemergence whole‐plant assays, dose–response experiments were conducted in all eight resistant and two susceptible accessions. Seeds were germinated in acrylic boxes (22 cm × 11 cm × 4.5 cm; Hoffman Manufacturing Inc., Corvallis, OR, USA), containing two blotter papers and 50 mL of distilled water, and incubated at 22 °C under a 12 h:12 h light/dark cycle and a photon flux density of 300 μmol m^−2^ s^−1^. After 7 days, seedlings at the BBCH‐10 stage (one‐leaf) were transplanted into 0.3‐L pots filled with the same sterilized soil and grown to a uniform height of approximately 2 cm. Indaziflam was applied using the same equipment and rates as in the preemergence assay. Survival was determined 8 weeks later, with live plants defined by green, actively growing tissue, and quantified by a binary variable (1 = survival, 0 = mortality).

#### 2.2.2 Temperature effects

Influence of temperature on resistance level was evaluated by both seed and preemergence whole‐plant assays using three resistant accessions (izr‐2, izr‐8, izr‐18) and two susceptible accessions (S‐ORG, S‐LB). Experiments were conducted under two climate‐controlled regimes representing seasonal variation in Oregon's Willamette Valley: a low‐temperature regime (9 °C day/1°C night) simulating January–February air temperature conditions, and a high‐temperature regime (25 °C day/12 °C night) simulating September–October. Light intensity was 100 μmol m^−2^ s^−1^. Seed viability was assayed by dose–response experiments (0.01 to 4.0 μm), a range selected from the initial resistance screening to capture responses from little inhibition at low doses to complete inhibition at high doses. Preemergence whole‐plant assays were conducted using fixed herbicide rates corresponding to estimates of lethal dose to 50% of the accession (LD_50_) and lethal dose to 90% of the accession (LD_90_) obtained from whole‐plant preemergence dose–response assays. The rates applied corresponded to 0.99 and 2.96 g ha^−1^ for S‐LB, 1.31 and 3.02 g ha^−1^ for S‐ORG, 3.36 and 9.75 g ha^−1^ for izr‐2, 10.6 and 35.8 g ha^−1^ for izr‐8, and 8.5 and 23.6 g ha^−1^ for izr‐18. Survival data were collected as in previous assays to determine whether resistance expression varied with temperature.

#### 2.2.3 Enzyme inhibitors

To investigate potential metabolic‐based resistance, seed assays were conducted in the presence of cytochrome P450 (CYP450) monooxygenases and glutathione S‐transferases (GST) inhibitors. These enzymes are among the most commonly implicated in herbicide metabolic detoxification in resistant weed populations.^25^ The CYP450 inhibitors included malathion^26^ (1 mm; Chem Service Inc., West Chester, PA, USA), tebuconazole^14^ (100 μm; Sigma‐Aldrich, St Louis, MO, USA), and 1‐aminobenzotriazole^27^, ^28^, ^29^ (1‐ABT, 1 mM; Sigma‐Aldrich), while the GST inhibitors were 4‐chloro‐7‐nitrobenzofurazan^30^ (NBD‐Cl, 250 μm; MP Biomedicals, Irvine, CA, USA) and ethacrynic acid^30^ (250 μm; Thermo Scientific, Waltham, MA, USA). Concentrations were based on previous studies and refined through preliminary testing to ensure they did not affect seed germination. These inhibitors are frequently used in herbicide resistance research to indirectly assess the role of metabolic pathways by observing whether herbicide efficacy improves when enzyme activity is suppressed. Malathion^26^, ^31^, ^32^ and 1‐ABT^14^, ^33^ are general CYP450 inhibitors that block monooxygenase activity, while tebuconazole^14^ is a triazole fungicide known to inhibit CYP450‐mediated demethylation processes. Ethacrynic acid^30^ and NBD‐Cl^30^ are commonly used to inhibit GST activity, which catalyzes the conjugation of glutathione to herbicide molecules, facilitating detoxification and sequestration.

Seeds from three resistant accessions (izr‐2, izr‐8, and izr‐18) and two susceptible lines (S‐ORG and S‐LB) were soaked in indaziflam solutions at concentrations corresponding to each accession's estimated herbicide rates LD_50_ and LD_90_, previously determined in seed‐based dose–response assays. The concentrations corresponded to 0.36 and 2.22 μm for izr‐2, 0.39 and 3.38 μm for izr‐8, 0.40 and 2.62 μm for izr‐18, 0.09 and 0.53 μm for S‐ORG, and 0.07 and 0.47 μm for S‐LB. Seeds were then transferred to Petri dishes containing solutions supplemented with distilled water (control) or known inhibitors of CYP450 or GSTs. Survival was scored after 30 days and compared to herbicide‐only treatments to evaluate whether inhibitors restored herbicide sensitivity.

#### 2.2.4 Multiple resistance screening

Resistance to other SOAs was assessed using postemergence and preemergence whole‐plant assays (Supporting Information Table S1). All eight resistant and two susceptible accessions were tested. Postemergence herbicides included glyphosate, a 5‐enolpyruvylshikimate‐3‐phosphate synthase (EPSPS) inhibitor (HRAC Group 9); rimsulfuron, an acetolactate synthase (ALS) inhibitor (Group 2); clethodim, an acetyl‐CoA carboxylase (ACCase) inhibitor (HRAC Group 1); flumioxazin, a protoporphyrinogen oxidase (PPO) inhibitor (HRAC Group 14); simazine, a photosystem II (PSII) inhibitor (HRAC Group 5); glufosinate, a glutamine synthetase inhibitor (HRAC Group 10); and pronamide, a microtubule assembly inhibitor (HRAC Group 3). Each herbicide was applied at 1× and 2× field rates to plants at the 2–3 leaf stage (BBCH‐23) (Table S1). Preemergence herbicides included dichlobenil, a CBI (HRAC Group 20); pendimethalin, a microtubule assembly inhibitor (HRAC Group 3); diuron, a PSII inhibitor (HRAC Group 7); flumioxazin, a PPO inhibitor (HRAC Group 14); napropamide, a very‐long‐chain fatty acid (VLCFA) elongase inhibitor (HRAC Group 15); pyroxasulfone, a VLCFA elongase inhibitor (HRAC Group 15); and fluridone, a carotenoid biosynthesis inhibitor (HRACGroup 12), all tested at recommended field rates. Plant regrowth and survival was assessed 8 weeks after treatment; individuals were rated as ‘alive’ if they had successfully germinated.

### 2.3 Field validation

Two field experiments were conducted from November 2022 to June 2023 in a commercial hazelnut orchard where resistant accession izr‐5 originated. Plants were at the BBCH‐10 growth stage and < 2.5 cm tall at the time of treatment application. Plots (2 m × 3 m) received early‐postemergence applications of indaziflam (50 and 100 g ha^−1^), glufosinate, or combinations of glyphosate with residual herbicides: indaziflam (50 or 100 g ha^−1^), dichlobenil, pendimethalin, diuron, flumioxazin, napropamide, pyroxasulfone, or fluridone (Table S1). Herbicides were applied using a carbon dioxide (CO_2_) pressurized backpack sprayer equipped with a spray boom containing three AI‐11002 nozzles (TeeJet Technologies) spaced at 50 cm and calibrated to deliver 187 L ha^−1^ at 275 kPa at 4.8 km h^−1^. The experiment employed a randomized complete block design with six replicates per treatment.

Control of *P. annua* was visually evaluated every 4 weeks after treatment, using a 0–100% scale, with 0% indicating no control and 100% representing complete control. Green coverage was assessed using two digital images per plot (iPhone 14), each covering approximately 6 m^2^. Images were analyzed with Canopeo software,^34^ using preset thresholds (cover crops foliage type, red‐to‐green ratio 1.00, blue‐to‐green ratio 0.80, minimum excess green 20). A subset of plots was visually validated to confirm software accuracy. At 28 weeks after treatment, aboveground biomass was sampled using two 0.25 m^2^ quadrats per plot, dried, and weighed.

### 2.4 Statistical analysis

Dose–response survival data were analyzed using a two‐parameter log‐logistic model (LL.2) with a binomial error distribution in R (version 4.3.1)^35^ using the drc package.^36^ The herbicide concentrations required to reduce survival by 50% (LD_50_) and 90% (LD_90_) were estimated for each accession. Pairwise comparisons of LD_50_ values were performed using the compParm() function, with statistical significance set at *P* < 0.05. When significant differences were detected, resistance factors (RFs) were calculated by dividing the LD_50_ of each resistant accession by that of the susceptible accessions. In this study, *P. annua* accessions were considered resistant if they exhibited LD_50_ or LD_90_ values at least two‐fold greater than those of the susceptible accessions.

Temperature experiments (whole‐plant assay only), enzyme inhibitor experiments, multiple resistance screening, and field experiments data were analyzed using analysis of variance (ANOVA). When significant treatment effects were detected (*P* < 0.05), Tukey's HSD (honestly significant difference) was used for pairwise comparisons.^37^

## 3 RESULTS

### 3.1 Resistance to indaziflam in *Poa annua* accessions

**Figure 2:**
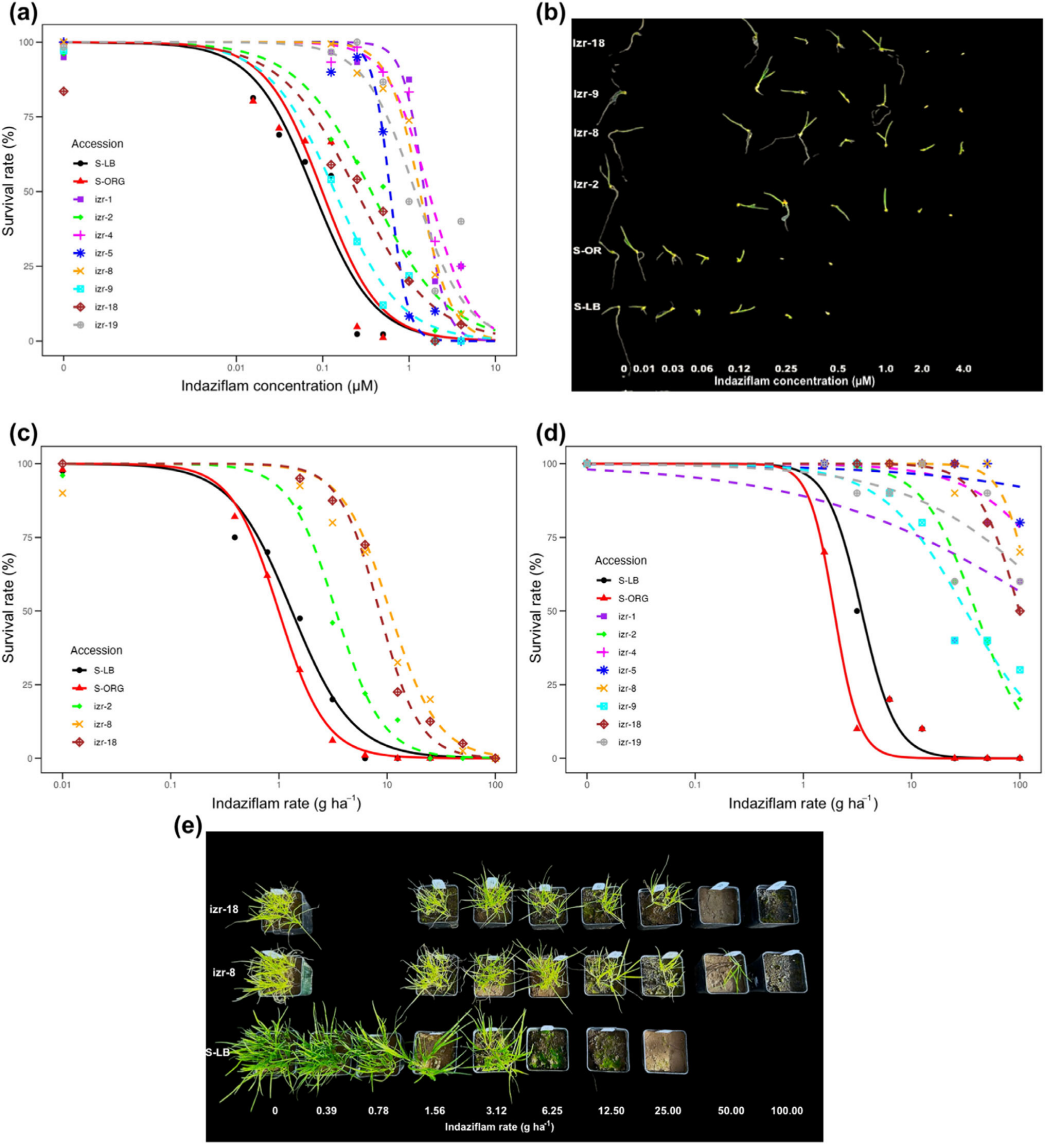
Dose–response analysis of *Poa annua* accessions to indaziflam. (a) Seed‐based dose–response curves showing survival (%) of eight resistant accessions and two susceptible accessions across increasing indaziflam concentrations (0–4.0 μm). (b) Representative seedling growth responses at 30 days after treatment from seed bioassays across indaziflam rates. (c) Preemergence whole‐plant dose–response curves for three resistant accessions (izr‐2, izr‐8, izr‐18) and two susceptible accessions (S‐ORG, S‐LB). (d) Early‐postemergence whole‐plant dose–response curves for the same ten accessions. (e) Whole‐plant survival and visual response 8 weeks after preemergence indaziflam application (0–100 g a.i. ha^−1^) in susceptible (S‐LB) and resistant (izr‐8, izr‐18) accessions. *n* = 6–8.

Seed‐based and whole‐plant dose–response assays confirmed indaziflam resistance in multiple *P. annua* accessions collected from Oregon hazelnut orchards (Table 2). In seed‐based assays (Fig. 2(a)), susceptible accessions S‐LB and S‐ORG exhibited LD_50_ values of 0.07 and 0.09 μm, respectively, and LD_90_ values of 0.47 and 0.53 μm. In contrast, resistant accessions had significantly higher LD_50_ values, ranging from 0.14 μm (izr‐9) to 1.69 μm (izr‐4), and LD_90_ values from 0.95 μm (izr‐9) to 5.73 μm (izr‐4). These differences correspond to RFs of 2‐ to 19‐fold relative to susceptible accessions.

**Table 2:** Dose–response parameter estimates and resistance factors for indaziflam of *Poa annua* accessions to indaziflam under seed‐based, preemergence, and early‐postemergence assays

Accession	Slope^†^	LD_50_ ^†^	LD_90_ ^†^	RF *versus* S‐LB^‡^	RF *versus* S‐ORG^‡^
*Seed‐based assay*		μm	μm		
S‐LB	1.23 (0.21)	0.07 (0.01)	0.47 (0.15)	—	—
S‐ORG	1.31 (0.26)	0.09 (0.01)	0.53 (0.16)	1.27 (ns)	—
izr‐1	3.52 (2.45)	1.50 (0.17)	2.81 (1.28)	19.23 (***)	15.13 (***)
izr‐2	0.99 (0.19)	0.37 (0.07)	3.34 (1.36)	4.66 (**)	3.70 (**)
izr‐4	1.80 (0.54)	1.69 (0.27)	5.73 (2.31)	21.68 (***)	17.12 (***)
izr‐5	4.25 (1.81)	0.60 (0.06)	1.01 (0.27)	7.76 (***)	6.10 (***)
izr‐8	2.36 (0.66)	1.33 (0.14)	3.38 (0.91)	16.99 (***)	13.49 (***)
izr‐9	1.14 (0.32)	0.15 (0.03)	0.95 (0.42)	2.14 (*)	1.40 (*)
izr‐18	0.99 (0.20)	0.25 (0.05)	2.26 (0.93)	3.14 (**)	2.49 (*)
izr‐19	1.47 (0.40)	1.18 (0.21)	5.23 (2.63)	13.89 (***)	9.73 (***)
*Preemergence assay*		g ha^−1^	g ha^−1^		
S‐LB	1.54 (0.26)	1.31 (0.16)	5.48 (1.35)	—	—
S‐ORG	1.96 (0.24)	0.99 (0.07)	3.00 (0.42)	0.76 (ns)	—
izr‐2	2.02 (0.27)	3.36 (0.23)	9.98 (1.67)	2.56 (***)	3.36 (***)
izr‐8	1.96 (0.39)	10.60 (1.21)	32.47 (8.31)	8.10 (***)	10.71 (***)
izr‐18	2.34 (0.55)	8.46 (0.79)	21.67 (5.16)	6.45 (***)	8.54 (***)
*Early‐postemergence assay*		g ha^−1^	g ha^−1^		
S‐LB	2.76 (0.80)	3.40 (0.40)	7.63 (2.19)	—	—
S‐ORG	3.76 (1.65)	1.93 (0.19)	3.46 (1.03)	0.56 (ns)	—
izr‐1	0.40 (0.16)	195.08 (73.27)	50 105 (15266)	56.64 (***)	101.29 (***)
izr‐2	1.77 (0.41)	39.16 (5.58)	135.48 (45.94)	11.37 (***)	20.33 (***)
izr‐4	1.04 (0.51)	343.74 (275.61)	2844 (5047)	99.81 (***)	178.48 (***)
izr‐5	0.39 (0.10)	54744 (8260)	151350 (21791)	15896 (***)	28425 (***)
izr‐8	3.17 (3.16)	133.37 (45.38)	266.61 (269.78)	38.73 (**)	69.25 (**)
izr‐9	1.12 (0.23)	31.77 (5.98)	226.06 (110.39)	9.23 (***)	16.50 (***)
izr‐18	2.28 (0.82)	98.72 (15.08)	258.88 (113.36)	28.67 (***)	51.26 (***)
izr‐19	0.63 (0.25)	262.08 (117.61)	8529.2 (18498)	76.10 (***)	136.08 (***)

^†^ Values are shown as mean with the standard error in parenthesis (*n* = 6–8).

^‡^ Resistance factors (RFs) were calculated as the ratio of LD_50_ for each izr accession to LD_50_ of the susceptible accessions (S‐LB or S‐ORG).: Significance levels for differences: ***: *P* < 0.001; **: *P* < 0.01; *: *P*< 0.05; ns: not significant. Pairwise comparisons of LD_50_ values were performed using the compParm() function in the R package drc.

In preemergence whole‐plant assays (Fig. 2(c)), LD_50_ estimates ranged from 3.3 g ha^−1^ (izr‐2) to 10.6 g ha^−1^ (izr‐8), compared to 0.99–1.31 g ha^−1^ in susceptible accessions (Table 2). LD_90_ estimates followed similar trends, with izr‐8 requiring 32 g ha^−1^ to achieve 90% control, or ten times higher than the rate for S‐ORG (3 g ha^−1^). All resistant accessions survived 25–63% at 25 g ha^−1^, and 13–25% survived at 50 g ha^−1^ (Table 1), indicating a high level of field‐relevant resistance.

Early‐postemergence whole‐plant assays reinforced these findings, revealing even greater resistance when indaziflam was applied postemergence rather than preemergence (Fig. 2(d)). For instance, the LD_50_ for izr‐5 exceeded the highest tested dose of 100 g ha^−1^, indicating a high level of resistance (Table 2). At the same time, izr‐1, izr‐4, and izr‐19 did not reach 50% mortality at the highest tested rate of 100 g ha^−1^, indicating that their LD_50_ values exceed this threshold and likely fall well beyond labeled field rates for indaziflam. In comparison, S‐LB and S‐ORG had LD_50_ values of 3.4 and 1.9 g ha^−1^, respectively. When indaziflam was applied at the one‐leaf stage (BBCH‐10), resistant accessions had LD_50_ values from 31 g ha^−1^ to greater than 100 g ha^−1^. At the highest tested rate (100 g ha^−1^), some resistant plants continued to grow and produce new tillers, indicating a severe loss of indaziflam efficacy under the postemergence application. Visual comparisons of seedling (Fig. 2(b)) and whole‐plant responses (Fig. 2(e)) corroborated these quantitative results, showing pronounced survival and growth in resistant accessions at herbicide doses that completely inhibited susceptible plants.

### 3.2 Low temperature enhances resistance expression

**Figure 3:**
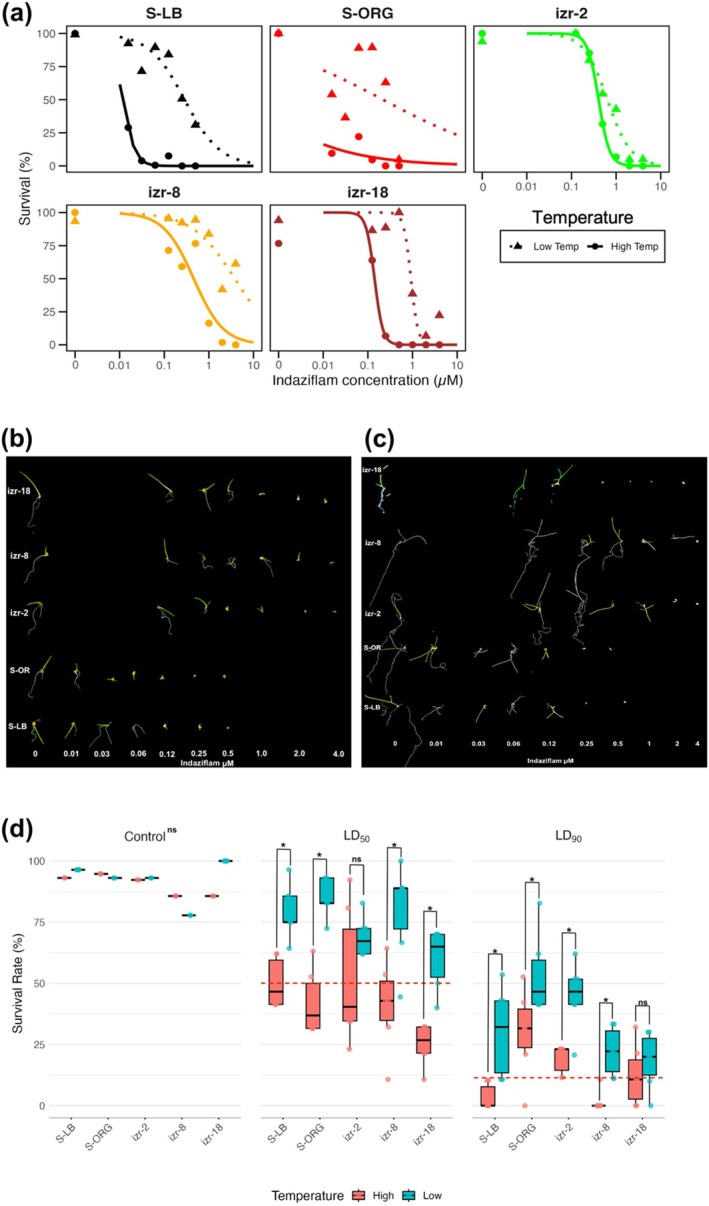
Effect of temperature on indaziflam resistance in *Poa annua* accessions. (a) Dose–response curves from seed‐based assays conducted under low (9 °C:1 °C; triangle and dotted line) and high (25 °C:12 °C; circle and solid line) temperature regimes, showing survival (%) of susceptible (S‐LB, S‐ORG) and resistant (izr‐2, izr‐8, izr‐18) accessions. (b, c) Representative images of seedling responses to increasing indaziflam concentrations under low (b) and high (c) temperature conditions for susceptible and resistant accessions. (d) Seedling survival at control, LD_50_, and LD_90_ rates from preemergence whole‐plant assays conducted under low (blue bars) and high (red bars) temperature regimes. LD_50_ values: S‐LB (1 g ha^−1^), S‐ORG (0.9 g ha^−1^), izr‐2 (4 g ha^−1^), izr‐8 (10 g ha^−1^), izr‐18 (6 g ha^−1^); LD_90_ values: S‐LB (3 g ha^−1^), S‐ORG (4 g ha^−1^), izr‐2 (10 g ha^−1^), izr‐8 (30 g ha^−1^), izr‐18 (23 g ha^−1^). Error bars represent standard error of the mean (*n* = 8); asterisks indicate significant differences between temperatures (*P* < 0.05); ns = not significant. Red dashed lines indicate reference thresholds corresponding to 50% (LD_50_) and 90% (LD_90_) survival for each accession.

Temperature influenced indaziflam sensitivity in both seed‐based and preemergence whole‐plant assays, with reduced herbicidal activity observed at lower temperatures (Table 3). Nevertheless, every resistant accession (izr‐1, izr‐4, izr‐5, izr‐19) survived all indaziflam doses at every temperature tested. Because their survival was independent of temperature, the resistance is best described as constitutive (expressed under all conditions examined) rather than conditional (expressed only under specific environmental circumstances). Under low‐temperature conditions (9 °C:1 °C), seed‐based LD_50_ values increased by two to eight times across all accessions compared to high‐temperature conditions (25 °C:12 °C), indicating stronger resistance expression at lower temperatures (Fig. 3(a)). For example, the LD_50_ for izr‐8 increased nearly eight‐fold under cooler conditions (0.46 μm at high temperatures *versus* 3.81 μm at low temperatures). Similar patterns were observed for izr‐2 and izr‐18 with two‐ to four‐fold increases in resistance. Susceptible accessions S‐LB and S‐ORG also survived greater indaziflam rates at low temperatures (e.g., S‐LB: 0.01 μm *versus* 0.29 μM), representing a 29‐fold increase in the amount of indaziflam required for control. Preemergence whole‐plant LD_50_ values followed a similar pattern (Fig. 3(d) and Table 3), with 1.3‐ to 2.4‐fold increases across all accessions under low temperatures compared to higher temperatures. For instance, izr‐8 survival increased from 41% at high temperatures to nearly 80% at its preemergence LD_50_ dose under low temperatures. This temperature effect was especially pronounced at LD_90_, where survival remained high in all accessions under cooler regimes but had higher mortality at high temperatures. All accessions had similar survival in the non‐treated control regardless of temperature regime, confirming that the observed effects were not due to differences in overall plant fitness or growth conditions.

**Table 3:** Dose–response parameter estimates and resistance factors for indaziflam of *Poa annua* accessions to indaziflam under high (25 °C:12 °C) and low (9 °C:1 °C) temperatures in seed‐based assays

Accession	Slope (high temperature)^†^	Slope (low temperature)^†^	LD_50_ (high temperature) (μm)^†^	LD_90_ (high temperature) (μm)^†^	LD_50_ (low temperature) (μm)^†^	LD_90_ (low temperature) (μm)^†^	Temperature effect (low *versus* high)^‡^
S‐LB	3.15 (2.88)	1.09 (0.42)	0.010 (0.005)	0.02 (0.01)	0.29 (0.07)	2.14 (1.90)	**
S‐ORG	0.39 (0.18)	0.31 (0.17)	0.002 (0.001)	0.04 (0.06)	0.22 (0.15)	2.65 (1.12)	*
izr‐2	3.53 (1.26)	1.65 (0.38)	0.40 (0.05)	0.75 (0.18)	0.64 (0.11)	2.42 (0.85)	*
izr‐8	1.29 (0.32)	0.91 (0.31)	0.46 (0.09)	2.50 (0.99)	3.81 (1.53)	42.82 (49.14)	*
izr‐18	4.64 (2.58)	5.45 (5.48)	0.14 (0.01)	0.23 (0.07)	0.92 (0.08)	1.38 (0.50)	***

^†^ Values are shown as mean with the standard error in parenthesis (*n* = 6–8).

^‡^ Temperature effect is the statistical comparison of LD_50_ between low *versus* high temperature within each accession. Significance levels for differences: ***: *P* < 0.001; **: *P* < 0.01; *: *P* < 0.05.

### 3.3 Metabolic inhibitors did not reverse resistance

**Figure 4:**
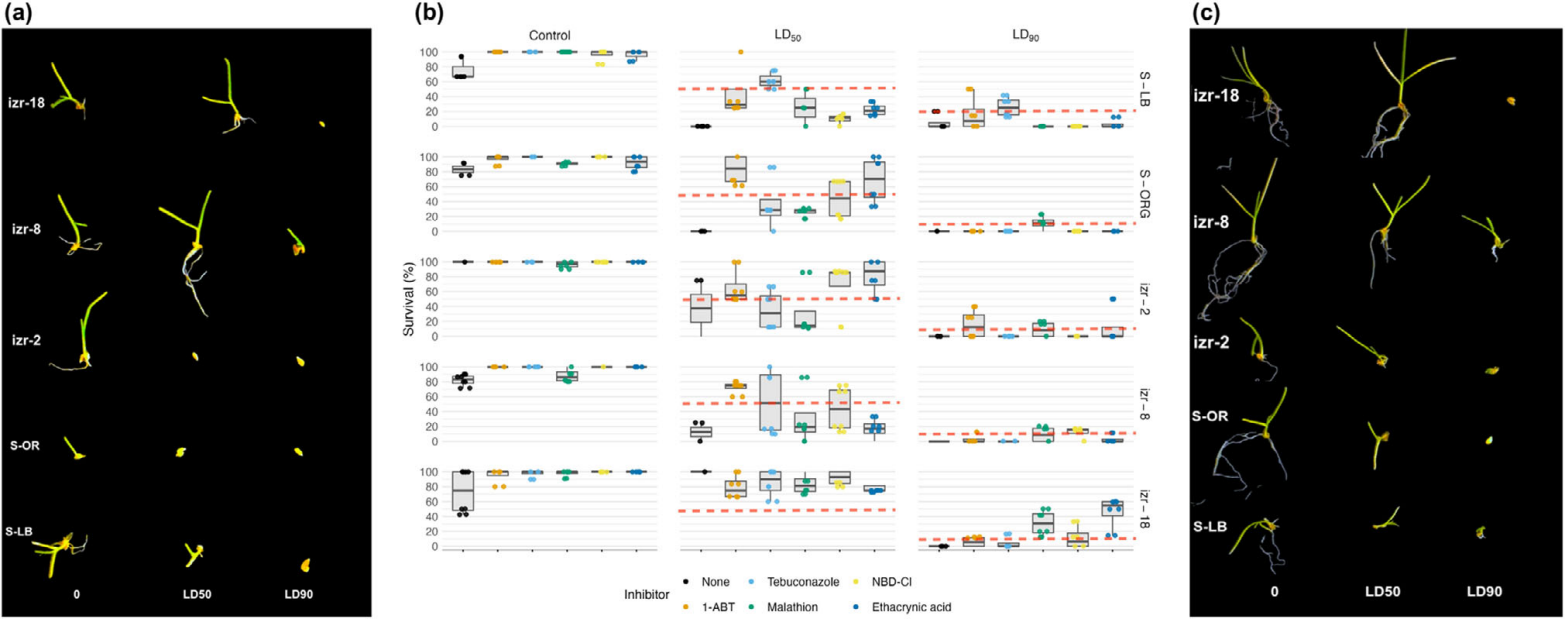
Visual comparison of seedling responses 30 days after seed bioassay establishment at 0 (non‐treated control), LD_50_, and LD_90_ concentrations, in the absence (a) or presence (c) of the cytochrome P450 inhibitor 1‐aminobenzotriazole (1‐ABT). (b) Seedling survival following indaziflam treatment at LD_50_ and LD_90_, with or without cytochrome P450 inhibitors (malathion, tebuconazole, 1‐ABT) or glutathione‐S‐transferase inhibitors (NBD‐Cl, ethacrynic acid). LD_50_ values: S‐LB (1 μm), S‐ORG (0.9 μm), izr‐2 (4 μm), izr‐8 (10 μm), izr‐18 (6 μm); LD_90_ values: S‐LB (3 μm), S‐ORG (4 μm), izr‐2 (10 μm), izr‐8 (30 μm), izr‐18 (23 μm). Box plots show the median, interquartile range, and whiskers, with individual points representing biological replicates (*n* = 6). Asterisks denote significant differences from the no‐inhibitor control (*P* < 0.05). Red dashed lines indicate reference thresholds corresponding to 50% (LD_50_) and 90% (LD_90_) survival for each accession.

*Poa annua* seeds were exposed to inhibitors of CYP450 (malathion, tebuconazole, and 1‐ABT) and GST (NBD‐Cl, ethacrynic acid) to test the involvement of metabolic detoxification pathways. None of the inhibitors significantly reversed resistance or consistently enhanced herbicide efficacy (Fig. 4). In several cases, seedlings treated with inhibitors exhibited statistically (*P* < 0.05) greater survival than those treated with herbicide alone. Visual phenotypes supported this trend: seedlings exposed to 1‐ABT showed better growth and reduced injury (Fig. 4(c)) than those in the water‐only treatment (Fig. 4(a)), suggesting potential antagonism rather than synergy.

### 3.4 Multiple herbicide resistance

**Figure 5:**
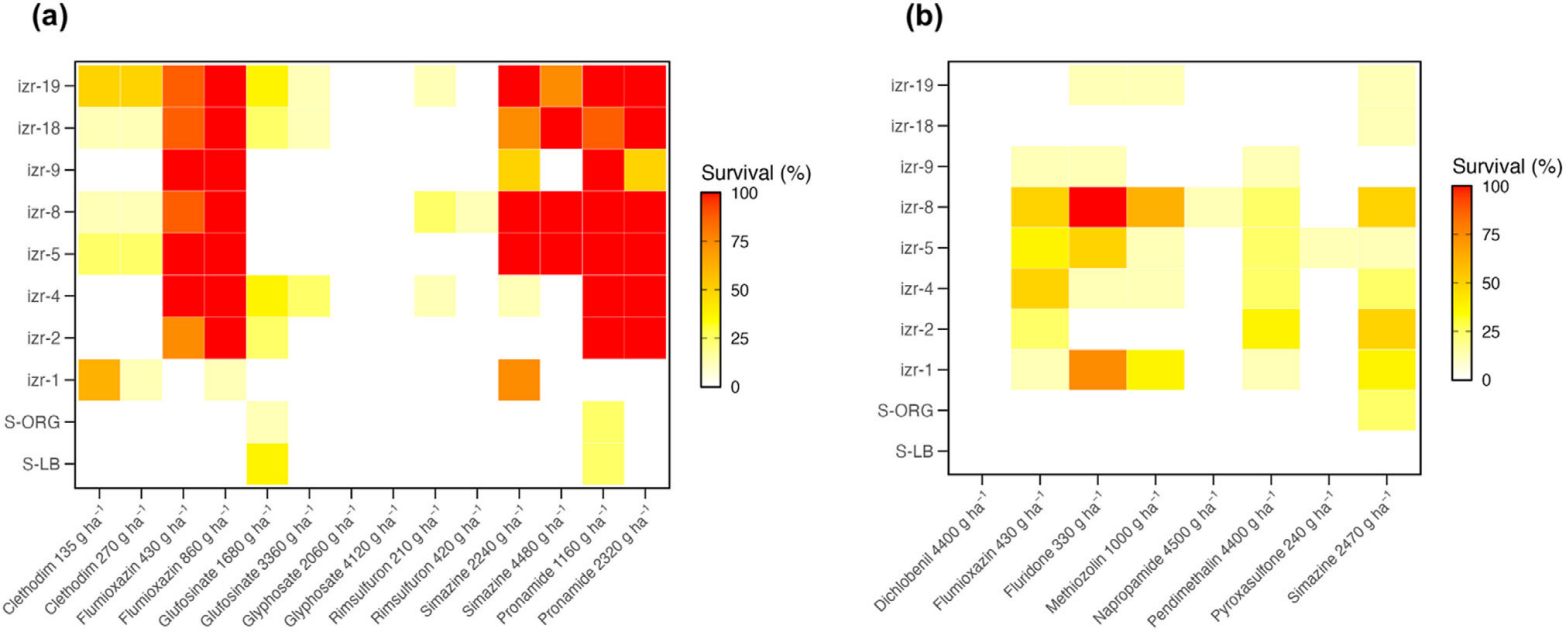
Heatmaps showing the survival rate (%) of *Poa annua* accessions in response to postemergence (a) and preemergence (b) herbicides 8 weeks after treatment. Each tile represents the mean survival for a given accession × herbicide combination. Survival rates are color‐coded from white (0%) to yellow (low survival rate), orange (moderate), and red (high), with red indicating poor herbicide efficacy, *n* = 8.

A glasshouse screening experiment using herbicides from 11 SOAs was conducted to evaluate multiple resistance patterns and identify potential control options. Overall, in postemergence treatments, resistant accessions exhibited high survival to rates of flumioxazin and pronamide alike, with most accessions showing > 75% survival (Fig. 5(a)). Survival to clethodim and simazine was accession‐dependent, with some resistant accessions like izr‐1 and izr‐19 exhibiting > 50% survival, with others exhibiting less than 25% survival. In contrast, rimsulfuron and glyphosate remained effective, with all resistant accessions showing < 13% survival. Preemergence herbicides varied in efficacy among resistant *P. annua* accessions (Fig. 5(b)). Fluridone applied preemergence was ineffective across several accessions, with izr‐1, izr‐2, izr‐5, and izr‐8 showing > 50% survival. Simazine, flumioxazin, methiozolin, and pendimethalin showed inconsistent performance, with survival ranging from 13% to 63%. In contrast, napropamide, dichlobenil, and pyroxasulfone provided greater control, resulting in < 13% survival. These findings indicate that resistance to indaziflam is often associated with resistance to multiple herbicide SOAs, significantly limiting chemical control options.

### 3.5 Field experiments validate seed‐assay and whole‐plant results

**Figure 6:**
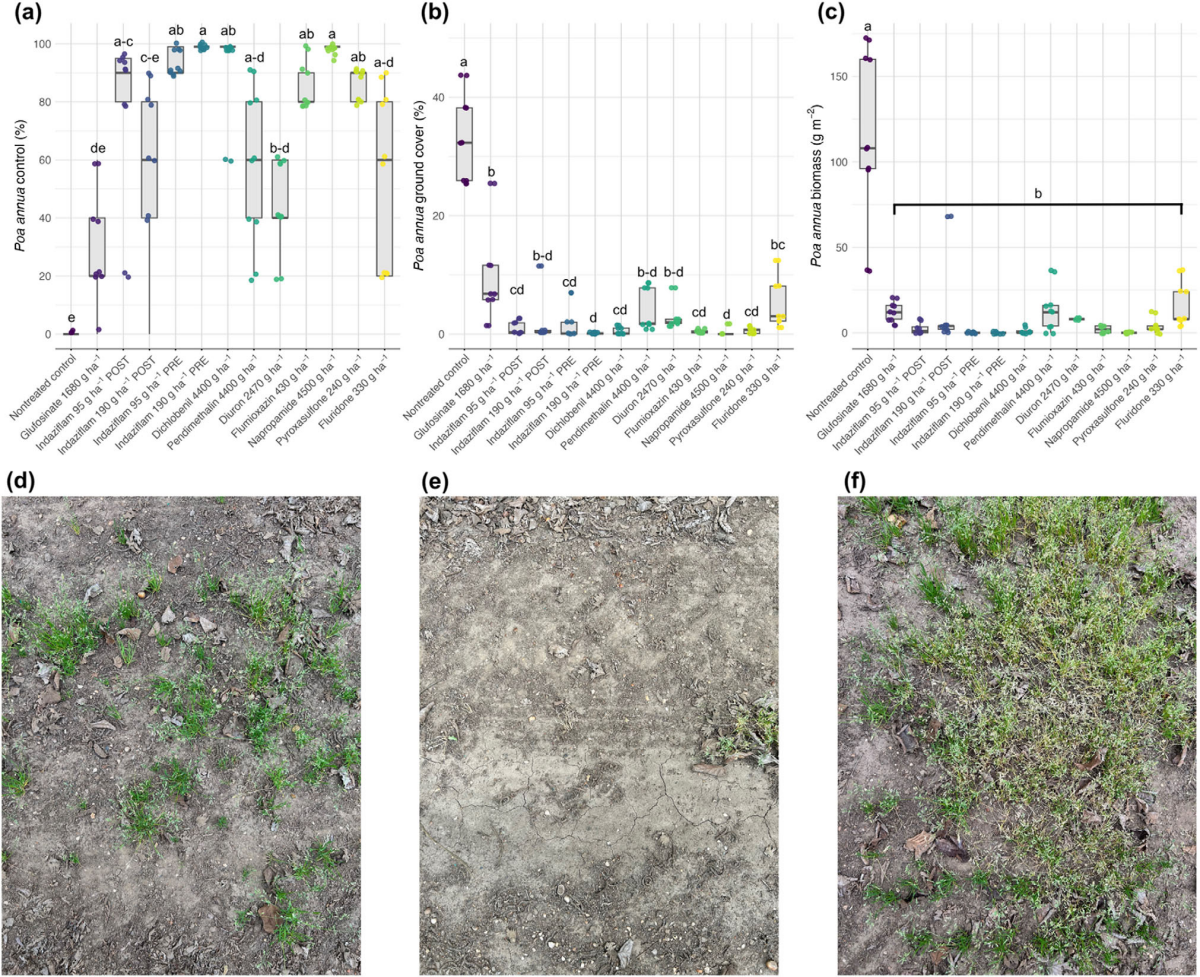
Field experiments in a hazelnut orchard infested with indaziflam resistant *Poa annua* (accession izr‐5), showing (a) visual control, (b) green ground cover estimated with Canopeo software, and (c) aboveground biomass at 28 weeks after treatment. Different letters indicate statistically significant differences among treatments (Tukey's HSD, *P* < 0.05). Representative field images illustrate *P. annua* growth under different treatments: (d) indaziflam early‐postemergence at 190 g ha^−1^, (e) indaziflam preemergence at 95 g ha^−1^, and (f) the non‐treated control.

Field experiments conducted in a commercial hazelnut orchard, where indaziflam‐resistant accessions were collected, generally supported glasshouse results and confirmed the limited efficacy of certain herbicides under field conditions, though with some variablity (Fig. 6). In terms of *P. annua* control (Fig. 6(a)), indaziflam applied preemergence at 95 and 190 g ha^−1^ provided > 85% control at 24 weeks after treatment, although with some variability and escapes were observed at the lower rate (Fig. 6(e)). In contrast, indaziflam applied postemergence (95 or 190 g ha^−1^) exhibited inconsistent control, with control values ranging from near 0% to 90%, and was not statistically different from the non‐treated control or treatment with glufosinate (Fig. 6(d),(f)). Glufosinate (1680 g a.i. ha^−1^) was also ineffective, with control statistically similar to the non‐treated control because of new *P. annua* emergence. Moreover, pendimethalin, diuron, and fluridone provided statistically similar control as glufosinate and postemergence‐applied indaziflam, indicating low and inconsistent performance of these herbicides.

This trend was reflected in the ground cover data (Fig. 6(b)), where the non‐treated control had the highest *P. annua* coverage, followed by glufosinate. Importantly, indaziflam at 190 g ha^−1^ applied postemergence, pendimethalin, diuron, and fluridone were not statistically different from glufosinate, indicating poor suppression with substantial *P. annua* survival. Biomass data further corroborated these findings (Fig. 6(c)). While all herbicide treatments significantly reduced biomass compared to the non‐treated control, there were no statistical differences among them, regardless of herbicide type or timing.

Although preemergence‐applied indaziflam was more effective than postemergence, variability and escapes suggest the presence of mixed resistant and susceptible individuals in the field. The relatively high orchard‐use rates (compared to glasshouse assays) likely suppressed many susceptible plants, but resistant individuals still survived, especially when applications were delayed until after emergence.

## 4 DISCUSSION AND CONCLUSION

This study provides additional confirmation of preemergence and early‐postemergence indaziflam resistance in *P. annua*,^14^, ^20^ highlighting the adaptability of *P. annua* under herbicide selection pressure.^5^, ^38^ We confirmed that resistance in three experimental systems: seed, whole‐plant, and field. Resistance was higher at low temperatures, reflecting the selection conditions for typical indaziflam use in cooler winter climates. Indaziflam‐resistant *P. annua* accessions, izr‐8 and izr‐18, had LD_90_ values exceeding 25 g ha^−1^ under preemergence conditions, equivalent to the lower rate in the range of field‐use rate. Under early‐postemergence conditions, resistance was even more pronounced, with LD_50_ values surpassing 100 g ha^−1^ in some accessions, demonstrating a significant shift in herbicide sensitivity.

Field experiments further generally validated glasshouse results, with control failures observed in the orchard from which accession izr‐5 was collected. Indaziflam applied early‐postemergence at 190 g ha^−1^ provided statistically similar control to glufosinate and the non‐treated control, confirming field‐level resistance. Even maximum label rate preemergence applications (95 g ha^−1^) showed inconsistent results, with escapes in several plants. The extended emergence window, high fecundity, and genetic plasticity of *P. annua* make it particularly prone to resistance evolution,^8^ and emphasize the importance of proactive, integrated management approaches. The observed resistance to indaziflam applied early‐postemergence in our study is consistent with prior reports in turfgrass systems.^14^ However, here we report evidence of both preemergence and early‐postemergence indaziflam resistance in *P. annua* unlike previous reports documenting either early post‐emergence^14^ alone or preemergence alone.^20^ The indaziflam resistance phenotype expressed preemergence and early‐postemergence suggests that resistant accessions can survive herbicide exposure at multiple developmental stages and may be difficult to control by altering application timing alone. Our results strongly support the resistance evolution framework proposed by Somerville *et al*.,^21^ which suggests that herbicides with long soil persistence and wide application windows, such as indaziflam, have greater risk for resistance evolution due to strong and prolonged selection pressure. The detection of both preemergence and early‐postemergence indaziflam resistance in *P. annua* not only supports this theory but also emphasizes that repeated use of persistent herbicides like indaziflam without rotation or complementary strategies can drive rapid resistance evolution.

The pronounced effect of temperature in modulating resistance level is particularly important for indaziflam, which is commonly applied to hazelnut orchards in winter. Our results showed that low temperatures (9 °C:1 °C) significantly increased survival in all accessions. Resistance was never eliminated, only more strongly expressed under cooler conditions, with LD_50_ values increasing by up to eight‐fold. Such findings are especially relevant for Oregon hazelnut production and other agroecosystems where indaziflam is applied during periods of low temperatures and rainfall. Temperature‐dependent variability in performance of foliar‐applied herbicide has been widely documented across multiple systems,^39^ and is often attributed to reduced uptake, translocation, or reduced metabolic rates that can lead to sublethal herbicide exposure, conditions known to favor the survival and selection of resistant biotypes.^40^, ^41^, ^42^ Our results reinforce this understanding, showing that even susceptible accessions exhibit increased tolerance under low temperatures. These results suggest that resistance to indaziflam in *P. annua* may have evolved in part due to selection pressure from applications made during cooler months, when indaziflam is typically used. Resistance mechanisms that function well under cold conditions are more likely to be favored in these situations, following a broader pattern seen in herbicide resistance, where resistance traits tend to perform best under the same environmental conditions in which they evolved.^43^, ^44^ This temperature sensitivity raises concerns about application timing, as selection pressure likely favored *P. annua* biotypes that thrive under cold conditions, because germination occurs during cooler months and because indaziflam is typically applied during that period. Herbicide applications under these conditions may underperform and unintentionally select for individuals with a higher natural tolerance or preexisting resistance alleles. Thus, while temperature and moisture influence herbicide performance, adjusting application timing alone may be insufficient to manage resistance. In this case, delaying application into colder periods may worsen control, as resistant individuals continue to emerge and postemergence applications become less effective.

The lack of resistance reversal with selected CYP450 or GST inhibitors, or by changes in temperature, suggests that enhanced metabolic degradation is unlikely to be the primary mechanism of indaziflam resistance in these accessions. In fact, in several cases, plant survival increased when inhibitors were applied after indaziflam treatment. Similar observations have been reported in *Lolium rigidum* Gaudin,^30^ where CYP450 or GST inhibitors failed to reverse resistance and frequently resulted in antagonistic effects. However, in our study, the inhibitors were not mixed with indaziflam, so we cannot attribute this effect to true antagonism. We also cannot rule out the possibility that the inhibitors were not specific to the enzymes involved in resistance, or that the application rates were too low to be effective. While metabolic detoxification is a common mechanism of resistance in many grass weed species,^25^, ^26^ our results suggest that resistance to indaziflam in these *P. annua* accessions may be mediated through an unknown mechanism. Examples of mechanisms are reduced uptake and/or translocation,^40^ sequestration,^2^ or mutations affecting target proteins. The unknown molecular target of indaziflam currently limits elucidation of this mechanism. However, recent research provides important clues. Huang *et al*.^45^ identified *Arabidopsis* mutants resistant to the cellulose biosynthesis inhibitor endosidin20, which carried mutations in the cellulose synthase A6 (CESA6) gene. These same mutations conferred cross‐resistance to indaziflam, suggesting potential overlap in herbicide action sites. Interestingly, despite targeting cellulose biosynthesis, dichlobenil remained effective across all resistant *P. annua* accessions in this study, suggesting that indaziflam resistance may not extend to all CBIs and supporting the possibility of a unique binding site or distinct mechanism. Previous work by Brosnan *et al*.^14^ showed higher foliar accumulation of indaziflam and its metabolites in *P. annua* plants resistant to early‐postemergence indaziflam, suggesting altered herbicide absorption or translocation. Further evidence comes from Reavell‐Roy,^46^ who identified weak indaziflam resistance in CULLIN1 (CUL1) mutant (izr‐1) *Arabidopsis* mutants, which exhibited phenotypic and physiological similarities to the auxin‐resistant cul1‐6 mutant, including increased root growth under low indaziflam concentrations. Their results suggested that CUL1 function, auxin signaling, and SCF‐mediated protein degradation contribute to indaziflam sensitivity, indicating multiple potential targets beyond cellulose biosynthesis. Collectively, these studies point to a multifaceted mode of action for indaziflam, potentially involving both cell wall biosynthesis and hormone‐regulated developmental processes. Further research employing transcriptomics, proteomics, and subcellular localization studies will be essential to elucidate the molecular mechanisms of indaziflam resistance.^47^ These tools may also enable the development of molecular diagnostics for early detection of resistance in the field, particularly important given the widespread resistance phenotypes now emerging.^48^

In addition to indaziflam, many accessions in this study exhibited poor control from herbicides with multiple SOAs, including PPO (flumioxazin), PSII (simazine, diuron), carotenoid biosynthesis (fluridone), fatty acid synthesis (methiozolin), and microtubule inhibitors (pendimethalin). Among the herbicides tested, only rimsulfuron and glyphosate applied postemergence, along with dichobenil, napropamide, and pyroxasulfone applied preemergence controlled all accessions, although their efficacy was also reduced in specific accessions. Notably, dichlobenil, like indaziflam, is a CBI, yet remained effective, suggesting that resistance to indaziflam may not confer cross‐resistance to all herbicides within the same mode of action. The presence of resistance to two or more SOAs in many accessions reduces the number of effective chemical control options and threatens the long‐term sustainability of chemical weed management in agroecosystems. Multiple resistance in *P. annua* is not unprecedented, where single accessions have already evolved resistance to as many as five or six SOAs.^13^, ^14^ It remains unclear whether this multiple resistance in our accessions arises from previous herbicide exposure or underlying shared resistance mechanisms, but they underscore the need for routine resistance screening and greater implementation of non‐chemical control tactics to preserve herbicide efficacy.

Our results provide strong and multifaceted evidence of indaziflam resistance in *P. annua*, affecting both preemergence and postemergence applications, and exacerbated by low temperatures. This resistance appears to be driven by an unknown mechanism. Sustaining the efficacy of indaziflam and similar herbicides will require a robust integrated weed management approach, incorporating diverse SOAs, non‐chemical strategies, and proactive resistance monitoring. Future research should prioritize understanding the genetic and physiological mechanisms of resistance, developing rapid detection tools, and evaluating the long‐term effectiveness of integrated control strategies under variable climatic and agronomic conditions.

## CONFLICT OF INTEREST

The authors declare no competing interests.

## Supporting information


**Table S1.** Herbicides, application rates, and adjuvants used in greenhouse and field experiments evaluating *Poa annua* resistance and control efficacy.

## Data Availability

The data that support the findings of this study are available from the corresponding author upon reasonable request.
