# Supplementary material for: Poa annua becomes the first weed to evolve resistance to indaziflam applied preemergence and early‐postemergence
Source: Pest Manag Sci. 2025 Sep 8;82(1):539–51. doi: 10.1002/ps.70214 (PMC12713700; doi:10.1002/ps.70214)
Supplement: Supplementary file 1 — Table S1. Herbicides, application rates, and adjuvants used in greenhouse and field experiments evaluating Poa annua resistance and control efficacy. [file PS-82-539-s001.docx]

**Short title:** Indaziflam resistance in *Poa annua*

***Poa annua* becomes the first weed to evolve resistance to indaziflam applied preemergence and early-postemergence**

Joshua W. A. Miranda^a,b,*^, Todd A. Gaines^c^, and Marcelo L. Moretti^b^

^a^Department of Horticulture, Michigan State University, East Lansing, MI 48824, USA.

^b^Department of Horticulture, Oregon State University, Corvallis, OR 97331, USA.

^c^Department of Agricultural Biology, Colorado State University, Fort Collins, CO 80523, USA.

***Corresponding author**: Joshua W. A. Miranda, [miran101@msu.edu](mailto:miran101@msu.edu)

JWAM: <https://orcid.org/0000-0003-3591-5190>

TAG: <https://orcid.org/0000-0003-1485-7665>

MLM: <https://orcid.org/0000-0001-8313-9195>

| **Table S1.** Herbicides, application rates, and adjuvants used in greenhouse and field experiments evaluating *Poa annua* resistance and control efficacy. | | | | |
| --- | --- | --- | --- | --- |
| Herbicide | Trade name | Rate | Adjuvant^†^ | Manufacturer and address |
|  |  | (g ha^-1^) |  |  |
| **Multiple resistance study – Postemergence** | | | | |
| Glyphosate | Roundup PowerMAX® 3 | 1680 (1X) | AMS + NIS | Bayer Crop Science, St. Louis, MO 63167 |
| Rimsulfuron | Matrix® SG | 210 (1X) | AMS + MSO | Corteva Agriscience™, Indianapolis, IN 46268 |
| Clethodim | Select Max® | 135 (1X) | AMS + MSO | Valent U.S.A. Corp., Walnut Creek, CA 94596 |
| Flumioxazin | Chateau® EZ | 430 (1X) | AMS + MSO | Valent U.S.A. Corp. |
| Simazine | Simazine 4L | 2240 (1X) | AMS + MSO | Drexel Chemical Company, Memphis, TN 38113 |
| Glufosinate | Rely 280® | 2060 (1X) | AMS | BASF Corp., Research Triangle Park, NC 27709 |
| Pronamide | Kerb® SC | 1160 (1X) | – | Corteva Agriscience™ |
| **Multiple resistance study – Preemergence** | | | | |
| Dichlobenil | CASORON® | 4400 | – | UPL Corporation, Cary, NC 27513 |
| Pendimethalin | Prowl® H2O | 4400 | – | BASF Corp. |
| Diuron | Diuron 4L | 2470 | – | Alligare, Opelika, Al 36801 |
| Flumioxazin | Chateau® EZ | 430 | – | Valent U.S.A. Corp. |
| Napropamide | DEVRINOL® 2-XT | 4500 | – | UPL Corporation |
| Pyroxasulfone | Zidua® SC | 240 | – | BASF Corp. |
| Fluridone | Brake® On! | 330 | – | SePRO Corporation, Carmel, Indiana 46032 |
| Methiozolin | PoaCure® | 1000 | – | Moghu Research Center Ltd., Gainesville, VA 20155 |
| **Field validation study** | | | | |
| Indaziflam | Alion® | 95 or 190 |  | Bayer Crop Science |
| Glufosinate | Rely 280® | 1,680 | AMS | BASF Corp. |
| Glyphosate | Roundup PowerMAX® 3 | 1,680 | AMS + NIS | Bayer Crop Science |
| Dichlobenil | CASORON® | 4400 | – | UPL Corporation |
| Pendimethalin | Prowl® H2O | 4400 | – | BASF Corp. |
| Diuron | Diuron 4L | 2470 | – | Alligare |
| Flumioxazin | Chateau® EZ | 430 | – | Valent U.S.A. Corp. |
| Napropamide | DEVRINOL® 2-XT | 4500 | – | UPL Corp. |
| Pyroxasulfone | Zidua® SC | 240 | – | BASF Corp. |
| Fluridone | Brake® On! | 330 | – | SePRO Corp. |
| ^†^Adjuvants: Ammonium sulfate (AMS; Amsol, WinField United, Arden Hills, MN, USA) was included at 10 g L⁻¹ , methylated seed oil (MSO; HASTEN-EA, Wilbur-Ellis, Aurora, CO, USA) was included at 8.9 g L⁻¹, or nonionic surfactant (NIS; Rainier®, Wilbur-Ellis) was included at 2.5 g L⁻¹ where noted. | | | | |
